# Editorial: Immune Control of JC Virus Infection and Immune Failure during Progressive Multifocal Leukoencephalopathy

**DOI:** 10.3389/fimmu.2017.01646

**Published:** 2017-11-27

**Authors:** Yassine Taoufik, Marie-Ghislaine de Goër de Herve

**Affiliations:** ^1^INSERM U1184, IMVA, Faculté de Médecine, Université Paris-Sud, Le Kremlin-Bicêtre, France

**Keywords:** JCV, progressive multifocal leukoencephalopathy, immunosuppression, biotherapies, T cells

**Editorial on the Research Topic**

**Immune Control of JC Virus Infection and Immune Failure during Progressive Multifocal Leukoencephalopathy**

From a scientific point of view, JCV is one of the most fascinating human viruses. This old, rustic, small, and stable DNA virus has cohabited with humans over the course of evolution (1), silently replicating in kidney epithelial cells and spreading from person to person through urine. Billions of people are infected worldwide, making JCV one of the most successful human viruses. Most remarkably, all this viral activity occurs with no detectable symptoms: even the primary infection appears to be clinically silent. JCV can elicit specific antibodies but its replication in the kidney does not appear to trigger effector T-cell responses (2), which appear more important than antibodies for controlling human polyomaviruses (Durali et al.). JCV replication in the kidneys thus appears to be perfectly tolerated by the human immune system, possibly as a result of long-term co-evolution driven by mutual benefit. However, contrasting with this idyllic picture of pacific and fruitful cohabitation with a discrete neighbor, JCV can on rare occasions become a merciless killer that targets the human central nervous system with lytic infection of glial cells, causing a rapidly fatal demyelinating disease called progressive multifocal leukoencephalopathy (PML). PML occurs almost exclusively in individuals with severe and prolonged immunosuppression that affects T-cells. PML, once a medical curiosity, entered the hall of infamy first during the AIDS pandemic and then as a major adverse effect of potent new immunosuppressive biologics such as natalizumab. AIDS, hematological malignancies, and natalizumab now account for more than 90% of all PML cases (3).

JCV infection is far from being fully understood, but several outstanding issues are addressed in a series of exciting papers published in this research topic.

Neurovirulent JCV strains that infect the brain are thought to arise from non-pathogenic “archetypal” viruses that replicate in the kidney, through a process of rearrangement (deletion–duplication) in the non-coding control region of the viral genome, as well as through mutations of the capsid protein VP1. From an evolutionary point of view, one might wonder why such a successful virus has this neurovirulent potential, which does nothing to promote its spread through the population, which is the driving force behind viral adaptation. JCV transmission may occur *via* the oral route (4–6). It is conceivable that this neurovirulence is a left-over property from an ancient period in which JCV transmission occurred mainly through brain-eating cannibalism, which appears to have been widespread within and between the different human subspecies that coexisted throughout prehistory (7–9). Even more speculatively, a predominantly neurotropic form might have co-evolved with *Homo-sapiens* to become the contemporary pacific archetypal virus. Other intriguing questions include whether or not other humanoid subspecies such as Neanderthals were susceptible to JCV and PML and, if so, whether this virus played a role in the extinction of these *Homo-sapiens* competitors.

Where and when neurotropic JCV variants are generated, and how they reach the brain, are controversial topics. One conventional view is that neurotropic viruses emerge after prolonged immunosuppression, then spread to the brain with the help of B-cells (Durali et al.). However, JCV has been detected in the brains of healthy individuals (10). JCV might, therefore, infect the brain of immunocompetent individuals but would remain under tight immunological control. As suggested by Frost and Lukacher, this control could involve brain-resident memory T-cells, which rely on help from the periphery, provided by re-circulating CD4-T-cells (Krzysiek et al.). This may explain why prolonged treatment with immunosuppressive antibodies such as natalizumab, which block T-cell trafficking, favor PML. JCV infection would thus represent a model of tissue-dependent immune tolerance, being tolerated in the kidney but under tight control in the brain. Frost and Lukacher stress that experimental models of polyomavirus CNS infection are urgently needed to understand the immunological mechanisms underlying PML.

Durali et al. discussed the role of B-cells as a reservoir and vector for JCV dissemination to the brain, along with the paradoxical and intriguing relationship between PML and rituximab, a B-cell-depleting monoclonal antibody. They suggest that rituximab may favor PML by disrupting the immunomodulatory effects of B-cells on T-cell responses. Indeed, accumulating evidence suggests that B-cells may exert important regulatory effects through cytokine secretion (Durali et al.).

Progressive multifocal leukoencephalopathy occurs following prolonged and profound systemic or cerebral T-cell immunosuppression. Yet, intriguingly, no more than 1% of patients who fulfill the necessary virological and immunological conditions actually develop PML (11, 12). In addition, this appears to be the case of both AIDS patients and patients on natalizumab (after a follow-up period of around 5 years), even though the underlying immunological mechanisms are distinct (11, 12). Hatchwell suspect genetic susceptibility to PML. Several rare primary immune deficits that may or may not be clinically apparent might favor PML onset when a secondary cause of prolonged immunosuppression occurs, such as immunosuppressive therapy, AIDS, or a hematological malignancy. If so, the immunological barrier to PML onset would be very high, requiring two distinct layers of immunosuppression. This could open the door to genetic tests for identifying patients at risk of PML during immunosuppressive treatments.

Natalizumab is one of the most effective treatments for relapsing–remitting multiple-sclerosis, but the risk of PML restricts its use. Monaco and Major and Antoniol and Stankoff describe research aimed at identifying biomarkers that could help mitigate the risk of PML. These authors emphasize the value of JCV serology but also its limitations, which include the risk of false-negative results (Monaco and Major; Antoniol and Stankoff). Both also mention the potential value of T-cell assays. However, while PML is a clearly defined disease in terms of its virological and neurological characteristics, its underlying immunological mechanisms are highly heterogeneous. Indeed, PML related to AIDS, hematological malignancies, natalizumab, B-cell-depleting antibodies such as rituximab, and drugs such as CTLA4 inhibitors that lift T-cell anergy (13) involves different immunological mechanisms, even if the consequences are the same, namely the inability to control JCV replication in the brain. While AIDS is associated with profoundly deficient CD4-help due to CD4-T-cell depletion, leading to impaired CD8-T-cell functions, there is no evidence that the anti-JCV immune response is intrinsically defective in patients on natalizumab. Rather, effector T-cells appear unable to cross the blood–brain barrier due to alpha4 integrin blockade, and this favors a state of local immunosuppression. Likewise, there is no evidence that natalizumab impairs the trafficking of antigen-presenting cells from the brain to peripheral lymph nodes. In natalizumab-treated patients with JCV replication in the CNS, dendritic cells coming from the brain might elicit anti-JCV T-cell effectors that cannot cross the blood–brain barrier but rather accumulate in the periphery and thus become detectable in blood with functional tests such as specific IFN-gamma release assay (2). In AIDS patients, these cells would only be detectable after a certain degree of immune recovery on antiretroviral treatment. This hypothesis is supported by the onset of immune reconstitution inflammatory syndromes (IRIS) following natalizumab withdrawal and plasma exchange, which was attributed to brain entry of both JCV and myelin-specific T-cell effectors that had previously accumulated in the periphery. While IRIS occurs in almost all MS patients on natalizumab, Fournier et al. report that this is only the case of 25% of AIDS patients with PML. These authors point out that IRIS is a complex phenomenon in terms of both its pathophysiology and its treatment, notably because the associated inflammation is also part of the immune response that controls JCV replication in the CNS (Fournier et al.).

I extend my warm thanks to all the authors of this outstanding PML Research Topic. Tremendous progress has been made since the emergence of PML in the wake of the AIDS pandemic, yet, many issues remain unresolved. It is likely that JCV was gradually tamed through co-evolution, but the recent increase in situations associated with immunosuppression has let the genie out of its bottle. There is an urgent need to identify patients at risk of PML, and for new therapeutic approaches to this devastating disease.

## Author Contributions

Both authors have made substantial, direct and intellectual contribution to the work, and approved it for publication.

## Conflict of Interest Statement

The authors declare that the research was conducted in the absence of any commercial or financial relationships that could be construed as a potential conflict of interest.

## References

[1] Agostini HT Yanagihara R Davis V Ryschkewitsch CF Stoner GL. Asian genotypes of JC virus in Native Americans and in a Pacific Island population: markers of viral evolution and human migration. Proc Natl Acad Sci U S A (1997) 94:14542–6. 10.1073/pnas.94.26.14542 9405649 PMC25048

[2] Hendel-Chavez H de Goër de Herve M-G Giannesini C Mazet A-A Papeix C Louapre C Immunological hallmarks of JC virus replication in multiple sclerosis patients on long-term natalizumab therapy. J Virol (2013) 87:6055–9. 10.1128/JVI.00131-13 23514886 PMC3648156

[3] Maillart E Taoufik Y Gasnault J Stankoff B Leucoencéphalopathie multifocale progressive. EMC (2017). 10.1016/S0246-0378(17)75522-4

[4] Bofill-Mas S Formiga-Cruz M Clemente-Casares P Calafell F Girones R. Potential transmission of human polyomaviruses through the gastrointestinal tract after exposure to virions or viral DNA. J Virol (2001) 75:10290–9. 10.1128/JVI.75.21.10290-10299.2001 11581397 PMC114603

[5] Bialasiewicz S Whiley DM Lambert SB Nissen MD Sloots TP. Detection of BK, JC, WU, or KI polyomaviruses in faecal, urine, blood, cerebrospinal fluid and respiratory samples. J Clin Virol (2009) 45:249–54. 10.1016/j.jcv.2009.05.002 19515607

[6] Vanchiere JA Nicome RK Greer JM Demmler GJ Butel JS. Frequent detection of polyomaviruses in stool samples from hospitalized children. J Infect Dis (2005) 192:658–64. 10.1086/432076 16028135 PMC4010313

[7] Mead S Stumpf MPH Whitfield J Beck JA Poulter M Campbell T Balancing selection at the prion protein gene consistent with prehistoric kurulike epidemics. Science (2003) 300:640–3. 10.1126/science.1083320 12690204

[8] Defleur A White T Valensi P Slimak L Crégut-Bonnoure E Neanderthal cannibalism at Moula-Guercy, Ardèche, France. Science (1999) 286:128–31. 10.1126/science.286.5437.128 10506562

[9] Rougier H Crevecoeur I Beauval C Posth C Flas D Wißing C Neandertal cannibalism and neandertal bones used as tools in Northern Europe. Sci Rep (2016) 6:29005. 10.1038/srep29005 27381450 PMC4933918

[10] White FA Ishaq M Stoner GL Frisque RJ. JC virus DNA is present in many human brain samples from patients without progressive multifocal leukoencephalopathy. J Virol (1992) 66:5726–34. 1326640 10.1128/jvi.66.10.5726-5734.1992 PMC241447

[11] Borchardt J Berger JR. Re-evaluating the incidence of natalizumab-associated progressive multifocal leukoencephalopathy. Mult Scler Relat Disord (2016) 8:145–50. 10.1016/j.msard.2016.03.005 27456891

[12] Engsig FN Hansen A-BE Omland LH Kronborg G Gerstoft J Laursen AL Incidence, clinical presentation, and outcome of progressive multifocal leukoencephalopathy in HIV-infected patients during the highly active antiretroviral therapy era: a nationwide cohort study. J Infect Dis (2009) 199:77–83. 10.1086/595299 19007313

[13] Dekeyser M de Goër de Herve M-G Hendel-Chavez H Labeyrie C Adams D Nasser GA Refractory T-cell anergy and rapidly fatal progressive multifocal leukoencephalopathy after prolonged CTLA4 therapy. Open Forum Infect Dis (2017) 4:ofx100. 10.1093/ofid/ofx100 28638849 PMC5473436

